# Supplementary material for: Marine Deep Biosphere Microbial Communities Assemble in Near-Surface Sediments in Aarhus Bay
Source: Front Microbiol. 2019 Apr 12;10:758. doi: 10.3389/fmicb.2019.00758 (PMC6474314; doi:10.3389/fmicb.2019.00758)
Supplement: Supplementary file 1 [file Data_Sheet_1.pdf]

Supplementary Material

Analysis of sediment cores from 2017 & 2018

In order to assess the reproducibility of the trends observed in our fine-scale analysis of a single core, four replicate sediment cores were taken from site M5 in Aarhus Bay during both 2017 and 2018. These cores were analyzed with a broader depth resolution than the core from 2014, focusing only on the sediment surface (1-2 cmbsf), the bottom of the bioturbation zone (5 cmbsf), and below (10-50 cmbsf) in 10 cm depth intervals. Within each sample, we analyzed (1) microbial activity (SRR), (2) microbial abundances (qPCR), and (3) community composition (Illumina MiSeq sequencing).

SRR and qPCR were both performed as described in the primary text, while sequencing of the 16S rRNA gene was performed with a modified protocol and a different sequencing technology. Bacteria and Archaea were sequenced separately, using the primer pairs Bac341F/ Bac805R (Herlemann et al., 2011) and Arch344Fmod/Arch915R (Casamayor et al., 2002; Xiao et al., 2017), respectively. Bacteria were sequenced in the core collected in 2017 and Archaea were sequenced in the core collected in 2018. Amplicon libraries for Bacteria and Archaea were prepared and sequenced according to Vergeynst *et al.* (2018) and Xiao *et al.* (2017), respectively. All libraries were sequenced on an Illumina MiSeq system at Aarhus University (Aarhus, Denmark). The raw sequencing reads were processed using the package DADA2 (v1.5.0) and the standard DADA2 workflow (Callahan et al., 2016) in R. Amplicon sequence variants (ASVs) were taxonomically classified in DADA2 using the SILVA SSU Ref v1.32 (Quast et al., 2013).

A					
**OTU**	**Phylum**	**Class**	**Order**	**Family**	**Genus**
**8**	Proteobacteria	Gammaproteobacteria	Chromatiales	Ectothiorhodospiraceae	*Acidiferrobacter*
**9**	Proteobacteria	Deltaproteobacteria	Myxococcales	Sandaracinaceae	unclassified
**36**	Proteobacteria	Deltaproteobacteria	Desulfobacterales	Desulfobulbaceae	*Desulfobulbus*
**11**	Bacteroidetes	BD2-2	unclassified	unclassified	unclassified
**100**	Proteobacteria	Deltaproteobacteria	Desulfobacterales	Desulfobacteraceae	Sva0081 sediment group
**39**	Proteobacteria	Deltaproteobacteria	Desulfuromonadales	Sva1033	unclassified
**82**	Proteobacteria	Gammaproteobacteria	Alteromonadales	Alteromonadaceae	OM60(NOR5) clade
**14**	Proteobacteria	Gammaproteobacteria	Chromatiales	Ectothiorhodospiraceae	*Acidiferrobacter*
**18**	Gemmatimonadetes	Gemmatimonadetes	PAUC43f_marine benthic_group	unclassified	unclassified
**19**	Actinobacteria	Acidimicrobiia	Acidimicrobiales	OM1_clade	unclassified
**B**					
**OTU**	**Phylum**	**Class**	**Order**	**Family**	**Genus**
**10**	Atribacteria	unclassified	unclassified	unclassified	unclassified
**2**	Proteobacteria	Deltaproteobacteria	Desulfarculales	Desulfarculaceae	unclassified
**41**	Actinobacteria	OPB41	unclassified	unclassified	unclassified
**43**	Planctomycetes	Phycisphaerae	MSBL9	unclassified	unclassified
**12**	Deinococcus-Thermus	Deinococci	Thermales	Thermaceae	*Oceanithermus*
**73**	Proteobacteria	Deltaproteobacteria	Desulfarculales	Desulfarculaceae	unclassified
**47**	Other	unclassified	unclassified	unclassified	unclassified
**132**	Nitrospirae	Nitrospira	Nitrospirales	Nitrospirales_Incertae Sedis	*Candidatus* Methylomirabilis
**20**	Spirochaetae	Spirochaetes	Spirochaetales	Spirochaetaceae	Termite Treponema cluster
**1**	Candidate_division_OP8	unclassified	unclassified	unclassified	unclassified

Table S1. Taxonomic identity of OTUs displayed in Figure 4. (a) OTUs that decrease in absolute abundance with sediment depth, displayed in Figure 4a. (b) OTUs that increase in absolute abundance with sediment depth, displayed in Figure 4b.

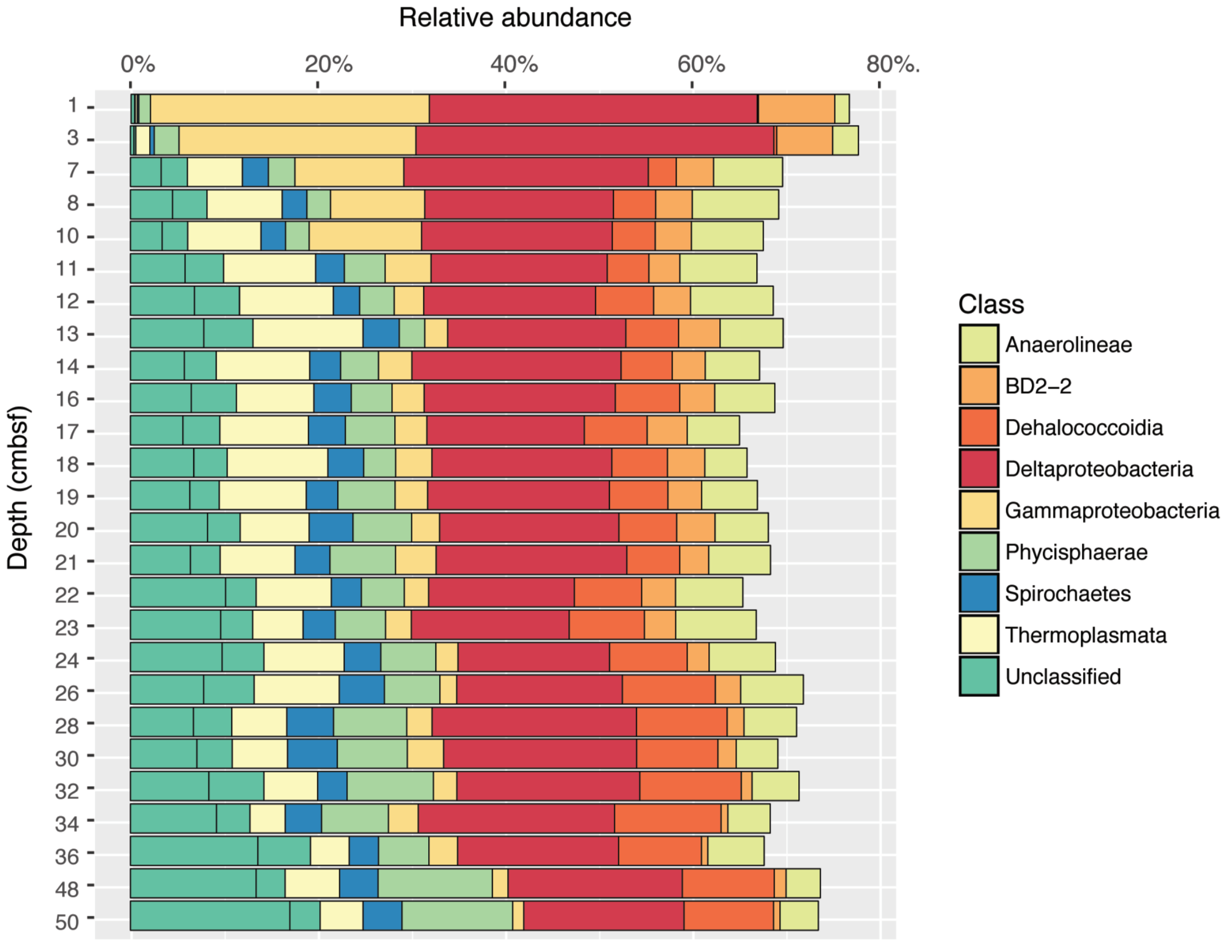

Figure S1. Relative abundance of the top 10 most abundant microbial classes identified by 16S rRNA gene sequencing. Colors correspond to the taxonomic identity at the class level and bar lengths represent the relative abundances of the classes out of the total number of sequencing reads at each depth.

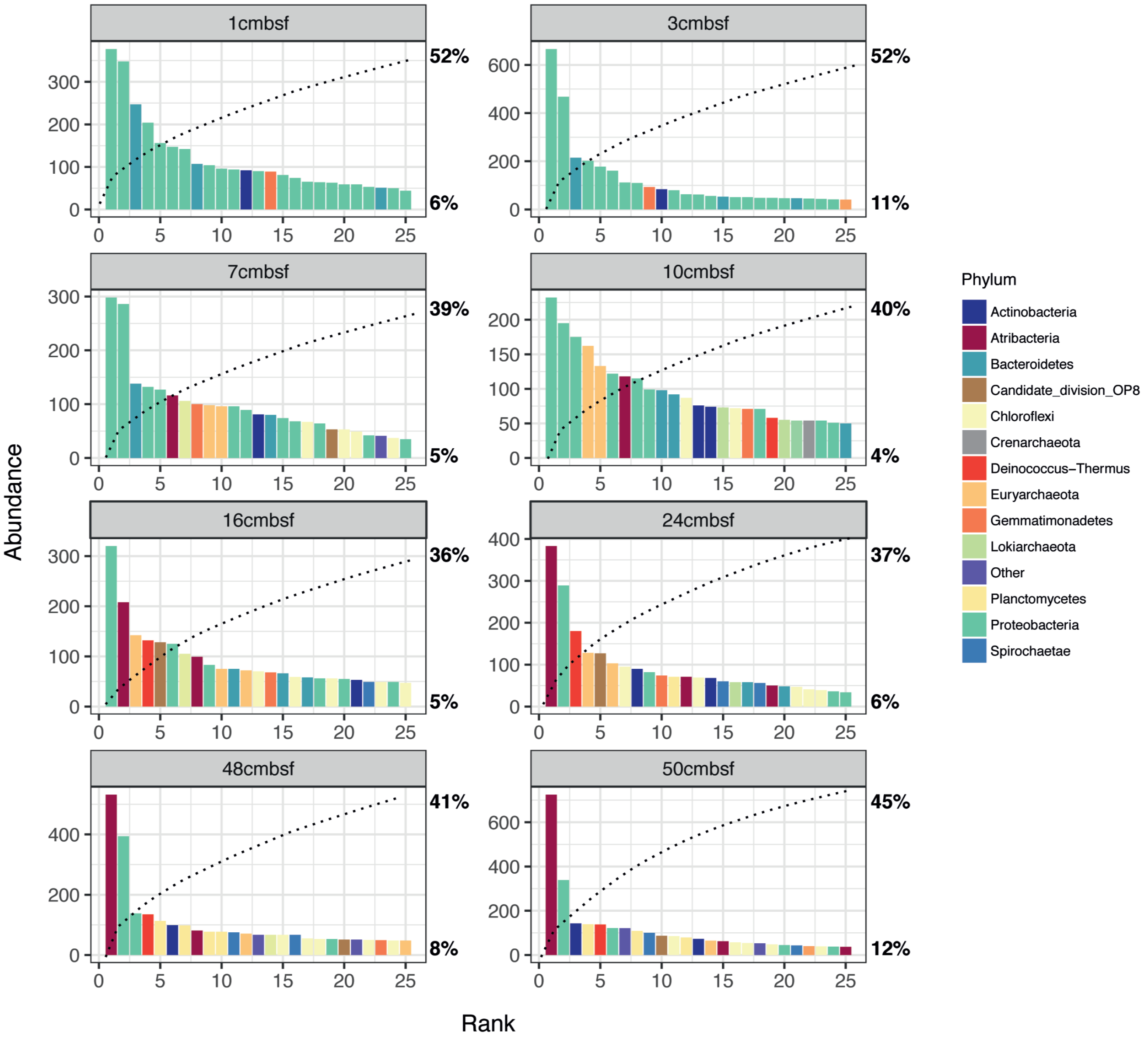

Figure S2. Rank abundance curves of the 25 most abundant OTUs identified by 16S rRNA gene sequencing throughout the sediment depth profile. Each panel displays the 25 most abundant OTUs at the indicated depth. The x-axis shows the rank of each OTU and the primary y-axis shows the number of sequencing reads assigned to each OTU. The colors of the bars indicate the taxonomic identity of each OTU on the phylum level. The dotted lines show the cumulative relative abundance (shown on the secondary y-axis) for the OTUs out of the total number of sequencing reads at that depth.

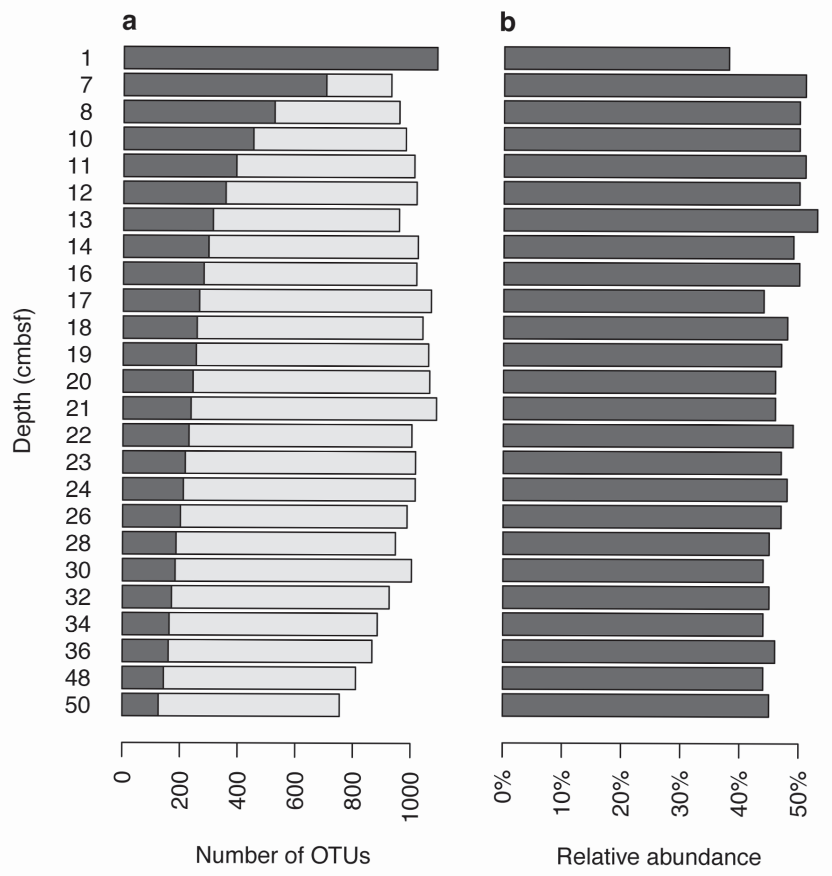

Figure S3. OTUs persisting across sediment depths. (a) Abundance of 16S rRNA gene sequence OTUs that persist across all depths in the sediment core. Light bars show the total number of OTUs present within a depth interval. Dark grey bars show the number of OTUs present within a depth that were also present in all of the above depths. (b) Relative abundance of persisting OTUs at each depth.

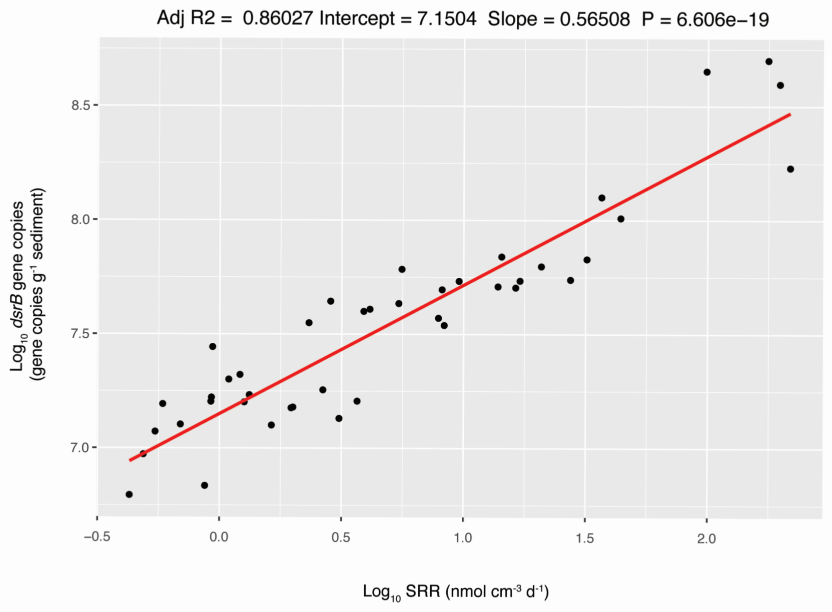

Figure S4. Correlation of *dsrB* gene copy numbers to measured sulfate reduction rates (SRR). P value is Pearson correlation coefficient. The correlation is based on comparing measured values for corresponding sediment depths between 1 and 50 cmbsf.

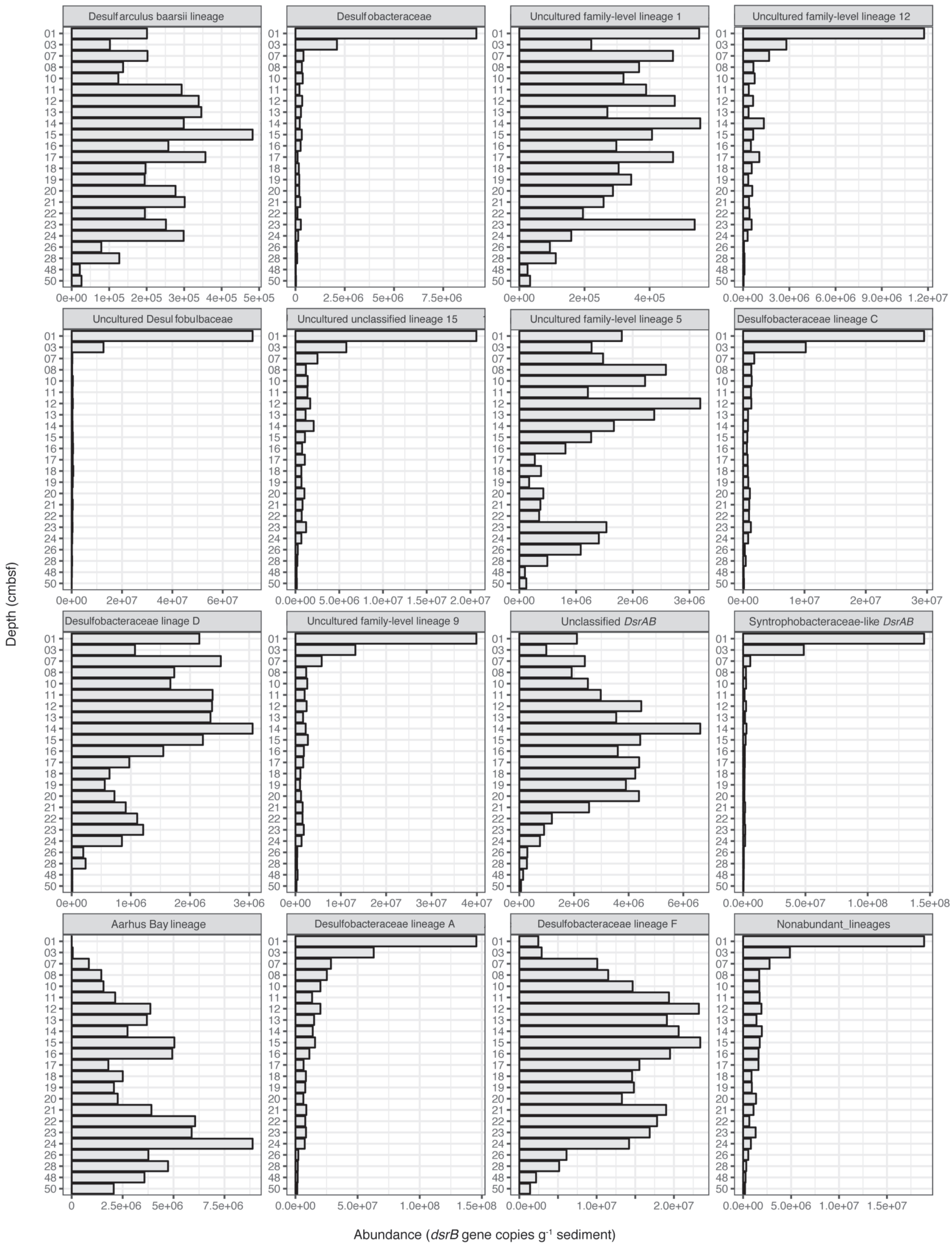

Figure S5. Absolute abundances of dominant lineages of sulfate reducing microorganisms (SRM). Absolute abundances were estimated by multiplying the relative abundances of *dsrB* gene sequences classified within each lineage by total *dsrB* gene copy numbers obtained from qPCR. Pooled nonabundant lineages comprised less than 20% of total sequences at each depth.

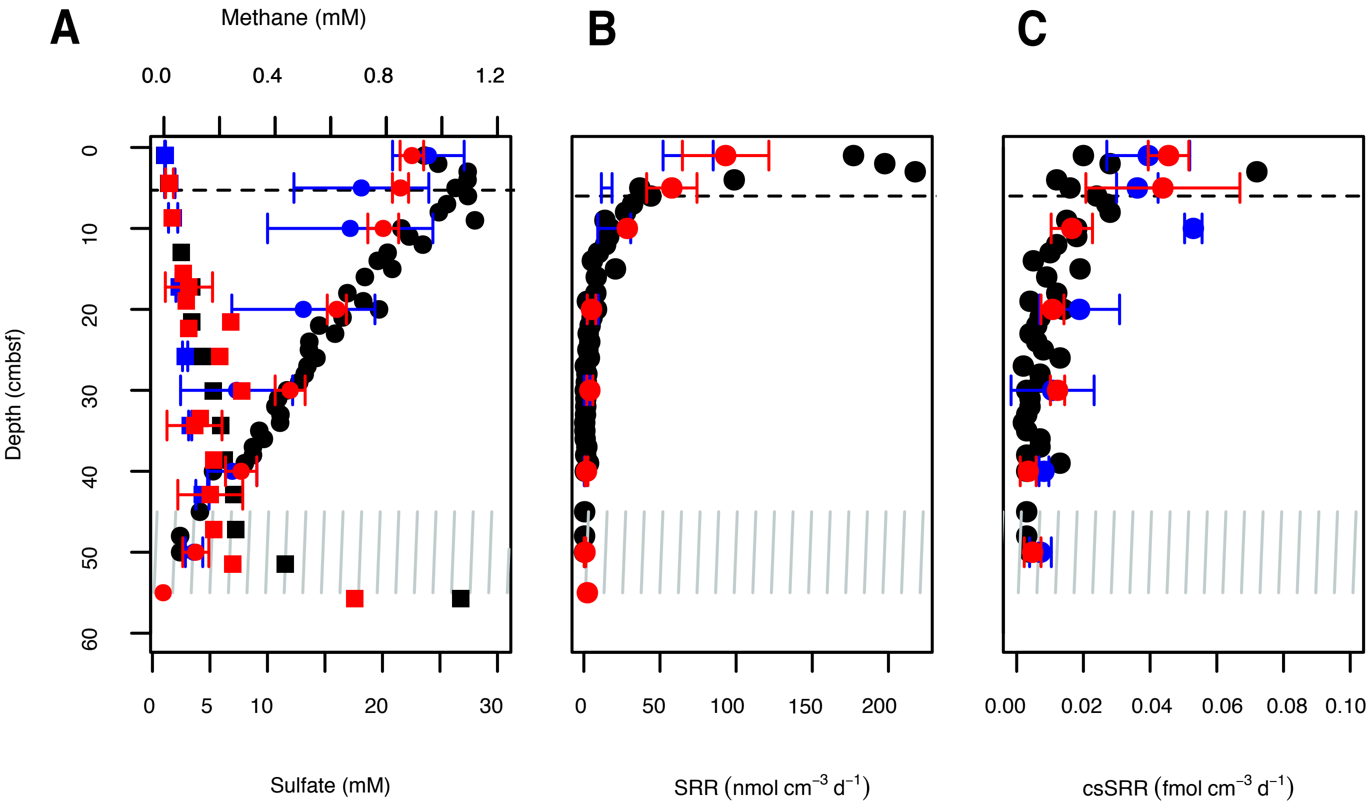

Figure S6. Spatiotemporal variation in (A) sediment porewater concentrations of sulfate and methane, (B) sulfate reduction rates (SRR), and (C) cell-specific sulfate reduction rates estimated using *dsrB* gene copy numbers as a proxy for SRM abundance. *DsrB* gene copy numbers are displayed in Figure S8. For (A) sulfate data are shown by circles and methane data are shown by squares. The color of the symbols refers to the sampling date, with cores taken from 2014 (black, n=1), 2017 (blue, n=4), and 2018 (red, n=4). The grey shaded region indicates the onset of the SMT.

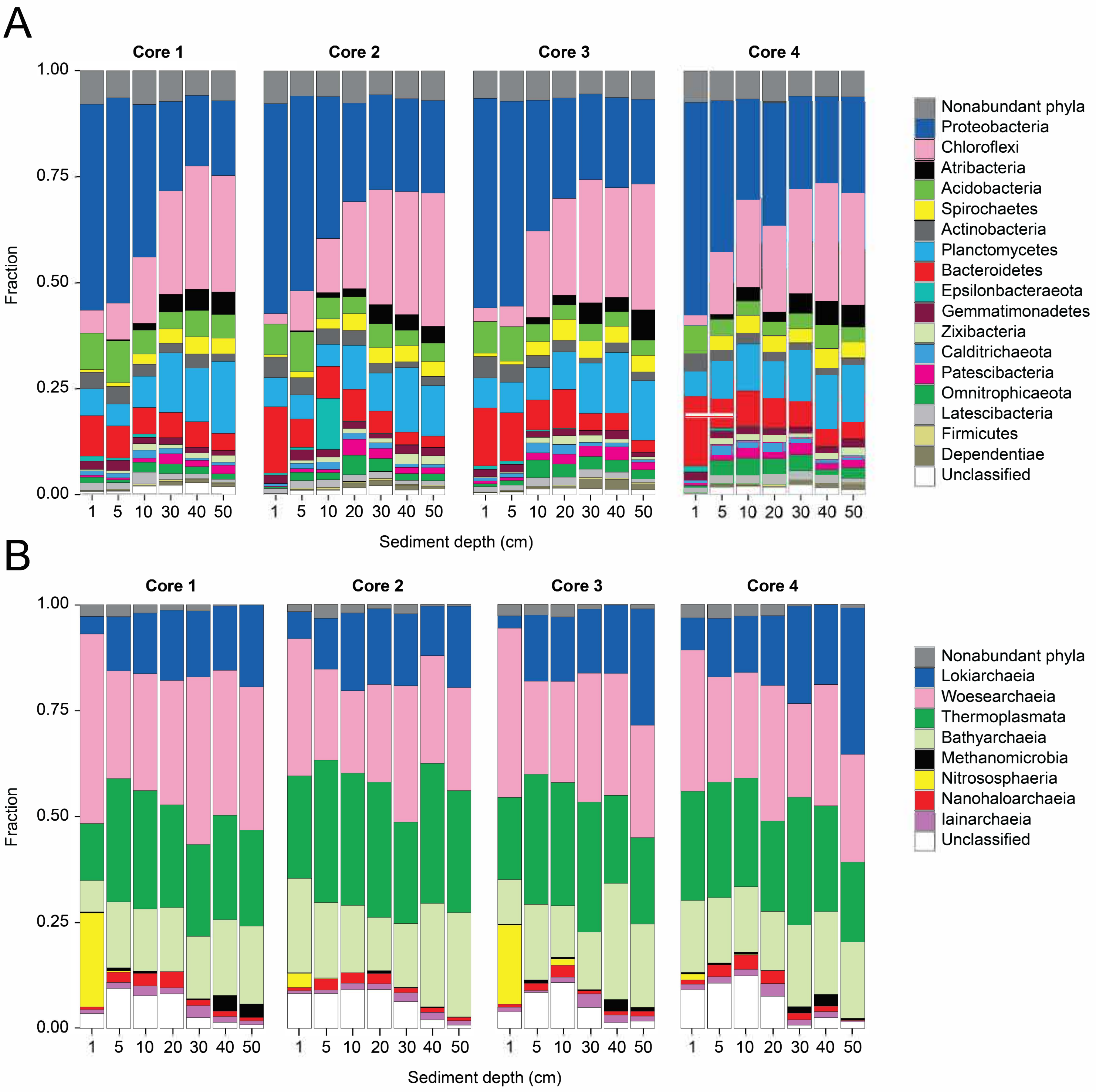

Figure S7. Microbial community composition within sediment cores sampled in 2017 and 2018. The stacked bar plots show relative abundances of taxonomic groups identified by 16S rRNA gene sequencing. (A) Relative abundance of Bacteria on the phylum level from four replicate cores collected in 2017. (B) Relative abundance of Archaea on the class level from cores collected in 2018.

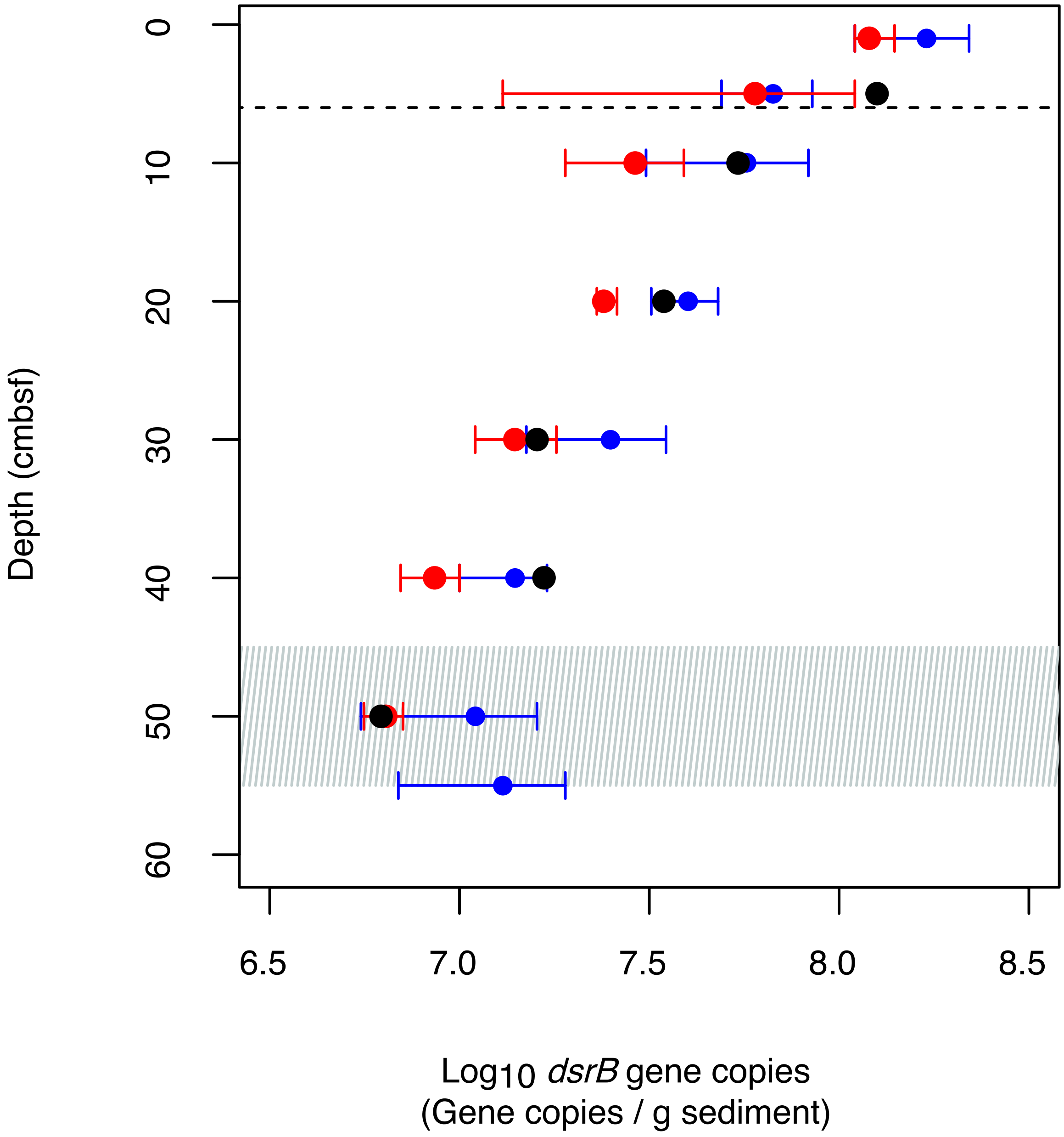

Figure S8. Spatiotemporal variation in abundance of SRM, as measured by qPCR of the *dsrB* gene. The color of the symbols refers to the sampling date, with cores taken from 2014 (black, n=1), 2017 (blue, n=4), and 2018 (red, n=4). The number of *dsrB* gene copies are presented on a log10 scale.

Supplementary References

Callahan, B. J., McMurdie, P. J., Rosen, M. J., Han, A. W., Johnson, A. J. A., and Holmes, S. P. (2016). DADA2: High-resolution sample inference from Illumina amplicon data. Nat. Methods. 13, 581-583. doi: 10.1038/nMeth.3869

Casamayor, E. O., Massana, R., Benlloch, S., Øvreås, L., Díez, B., Goddard, V. J., Gasol, J. M., Joint, I., Rodríguez-Valera, F., and Pedrós-Alió, C. (2002). Changes in archaeal, bacterial, and eukaryal assemblages along a salinity gradient by comparison of genetic fingerprinting methods in a multipond solar saltern. *Environ. Microbiol.* 4, 338-348. doi: 10.1046/j.1462-2920.2002.00297.x

Herlemann, D. P. R., Labrenz, M., Jürgens, K., Bertilsson, S., Waniek, J. J., and Andersson, A. F. (2011). Transitions in bacterial communities along the 2000 km salinity gradient of the Baltic Sea. *ISME J.* 5, 1571-1579. doi: 10.1038/ismej.2011.41

Quast, C., Pruesse, E., Yilmaz, P., Gerken, J., Schweer, T., Yarza, P., Peplies, J., and Glöckner, F. O. (2013). The SILVA ribosomal RNA gene database project: improved data processing and web-based tools. *Nucleic Acids Res.* 41, D590-D596. doi: 10.1093/nar/gks1219

Vergeynst, L., Kjeldsen, K. U., Lassen, P., and Rysgaard, S. (2018). Bacterial community succession and degradation patterns of hydrocarbons in seawater at low temperature. *J. Hazard. Mater.* 353, 127-134. doi: 10.1016/j.jhazmat.2018.03.051

Xiao, K., Beulig, F., Kjeldsen, K. U., Jørgensen, B. B., and Risgaard-Petersen, N. (2017). Concurrent methane production and oxidation in surface sediment from Aarhus Bay, Denmark. *Front. Microbiol.* 8:1198. doi: 10.3389/fmicb.2017.01198
